# Advances in 16S rRNA‐Based Microbial Biomarkers for Gastric Cancer Diagnosis and Prognosis

**DOI:** 10.1111/1751-7915.70115

**Published:** 2025-02-24

**Authors:** Yingying Wang, Xunan Qiu, Aining Chu, Jijun Chen, Lu Wang, Xiaohu Sun, Bengang Wang, Yuan Yuan, Yuehua Gong

**Affiliations:** ^1^ Tumor Etiology and Screening Department of Cancer Institute and General Surgery The First Hospital of China Medical University Shenyang China; ^2^ Key Laboratory of Cancer Etiology and Prevention in Liaoning Education Department The First Hospital of China Medical University Shenyang China; ^3^ Key Laboratory of GI Cancer Etiology and Prevention in Liaoning Province The First Hospital of China Medical University Shenyang China

**Keywords:** 16S rRNA gene sequencing, clinical application, gastric cancer, microbial biomarkers, microbiota

## Abstract

Gastric cancer (GC) is a malignant tumour with high morbidity and mortality worldwide, and there is an urgent need for early diagnosis and precision treatment. In recent years, the role of microbiota in the occurrence and development of GC has drawn extensive attention. Particularly, the in‐depth study of GC‐related microbiota by 16S rRNA sequencing technology has offered valuable tools and new perspectives for exploring the microbial characteristics of GC patients. This review systematically summarises the microbial diversity and composition of GC and non‐GC samples based on 16S rRNA data, outlines the progress in identifying GC‐related microbial biomarkers, explores the potential mechanisms by which diagnostic microbial biomarkers influence the development of GC, and reflects on the limitations of present studies. By integrating the current evidence, this review intends to offer a new theoretical foundation and further direction for the clinical translation of microbiota research in the diagnosis and treatment of GC.

## Graphical abstract

This review summarises the microbial diversity and composition in gastric cancer (GC) and non‐GC samples based on 16S rRNA data, outlines GC‐related microbial biomarkers, explores their role in GC development, and reflects the limitations of current research. It intends to provide a foundation for clinical microbiota research in GC diagnosis and treatment.

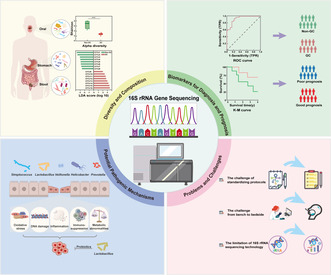

## 1 Introduction

Gastric cancer (GC) remains a significant public health challenge worldwide. In 2022, the International Agency for Research on Cancer reported approximately 968,000 new cases and 660,000 deaths globally, ranking GC as the fifth most common cancer in terms of both incidence and mortality (Bray et al. 2024). The numbers are projected to increase by 66% in incidence and 71% in mortality comparing between years 2020–2040 (Morgan et al. 2022). These alarming trends highlight the urgent need for improved prevention, early detection, and treatment strategies. Since the first isolation of *Helicobacter pylori* ( *H. pylori* ) from gastric mucosa in 1982, research has progressively uncovered the role of the microbiota in gastric carcinogenesis (Warren and Marshall 1983; Bik et al. 2006; Coker et al. 2018; Ferreira et al. 2018). These findings underscore the importance of further investigating microbial contributions to GC development.

For years, the limitations of molecular biology techniques have impeded a comprehensive understanding of the characteristics of the human gastrointestinal microbiota. However, recent advancements in gene‐based, culture‐independent microbial analysis methods, particularly 16S ribosomal RNA (rRNA) gene sequencing, have overcome these challenges. This method, which examines both conserved and variable regions (such as the V4 region) of the 16S rRNA gene, has become essential for taxonomic classification of bacterial genomes and the basis of bacterial diversity research (Lane et al. 1985; Olsen et al. 1986). Leveraging this technique, researchers have made significant progress in characterising the microbiota associated with GC. Although several recent reviews have comprehensively summarised the role of the microbiota in GC progression, these insights significantly enhance our understanding of the microbiota's involvement and its underlying mechanisms in the pathogenesis of GC (Guo et al. 2022; Liao et al. 2023; Wang et al. 2023). Relatively speaking, however, they seem to overlook the clinical relevance of microbial taxonomic biomarkers in GC diagnosis, treatment response, and prognosis. To the best of our knowledge, no reviews to date have systematically addressed the potential of 16S rRNA‐based microbial biomarkers within these fields, which would be very valuable for their clinical applications.

**FIGURE 1:**
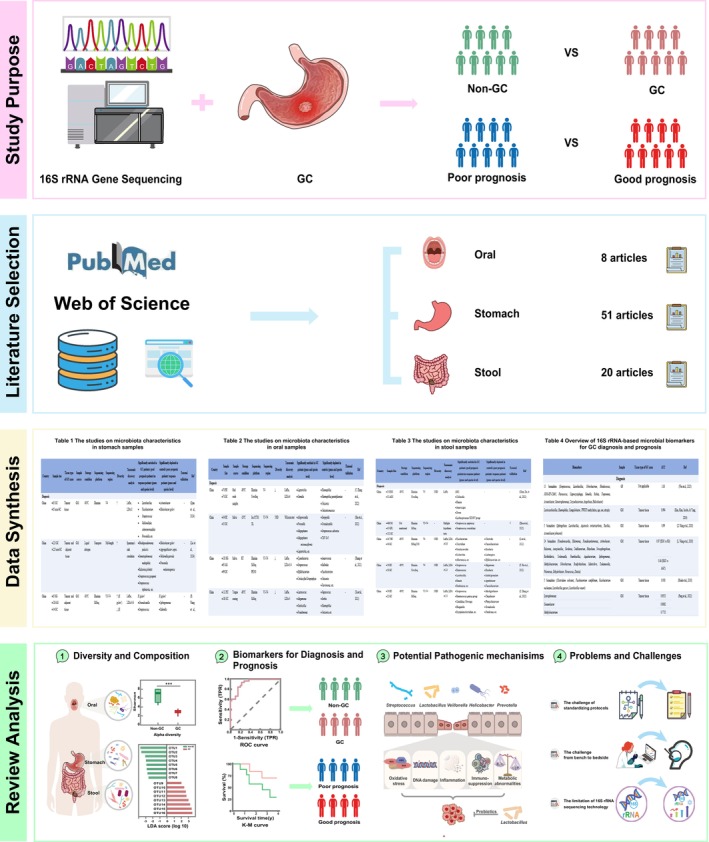
Overview of 16S rRNA‐based microbial biomarkers in GC diagnosis and prognosis. A general overview of this study, summarising its purpose, literature selection, data synthesis, and review analysis of 16S rRNA‐based microbiota research in GC.

Therefore, to fill this gap, we systematically searched for literature published over the past decade on PubMed (https://pubmed.ncbi.nlm.nih.gov/) and Web of Science (https://www.webofscience.com/) related to microbiota and GC, using the keywords ((stomach cancer) OR (gastric cancer)) AND (microb*)) AND (16S rRNA sequencing)). Then we presented a comprehensive overview of 16S rRNA‐based microbial characteristics in various types of samples and evaluated the potential of microbial biomarkers for GC diagnosis and prognosis, as well as the underlying mechanisms. Additionally, we analysed the current challenges faced in microbiota –GC research, offering insights that may guide more precise, microbiota‐centred approaches aimed at enhancing GC diagnosis and treatment (Figure 1).

## 2 16S rRNA‐Based Microbial Diversity and Composition in GC From Multiple Sample Sources

### 2.1 16S rRNA‐Based Microbial Diversity and Composition in GC From Stomach Samples

Stomach samples, including gastric mucosa (GM) and gastric fluid (GF), have been extensively studied to investigate microbial diversity and composition. A summary of recent research is provided in Table 1.

**TABLE 1:** The studies on microbiota characteristics in stomach samples from GC patients based on 16S rRNA gene sequencing.

Country	Sample size	Tissue type of GC cases	Sample source	Storage condition	Sequencing platform	Sequencing region	Diversity	Taxonomic discovery analysis	Significantly enriched in GC patients/good prognosis patients/no response patients (genus and species level)	Significantly depleted in controls/poor prognosis patients/response patients (genus and species level)	External validation	Ref
*Diagnosis*												
China	85 GC 85 non‐GC	Tumour tissue	GM	−80°C	Illumina	V4	↑	LefSe, LDA > 2	*Lactobacillus* *Fusobacterium* *Streptococcus* *Veillonellales selenomonnadales* *Prevotella*, etc.	*Acinetobacter* *Helicobacter pylori*	—	(Qian et al. 2024)
China	223 GC 223 non‐GC	Tumour and adjacent tissue	GM	Liquid nitrogen	Nanopore	Full‐length	↑	Spearman's rank correlation	*Rhodopseudomonas palustris* *Stenotrophomonas maltophilia* *Ralstonia pickettii* *Streptococcus pyogenes* *Streptococcus infantarius* , etc.	*Helicobacter pylori* *Aggregatibacter segnis* *Veillonella parvula* *Prevotella melaninogenica*	—	(Lei et al. 2024)
China	63 SG 126 AG 14 GC	Tumour and adjacent tissue	GM	−80°C	Illumina MiSeq	V3–V4	↑ ( *H. pylori* ^−^) ↓ ( *H. pylori* ^−+^)	LefSe, LDA > 3.6	*H. pylori* ^*−*^ *Granulicatella* *Streptococcus* *H. pylori* ^+^ *Actinobacillus* *Haemophilus* *Neisseria* *Streptococcus*	*H. pylori* ^*−*^ *Sphingomonas* *Rahnella* *Rumihococcus* *Serratia* *Pseudomonas* *H. pylori* + *Helicobacter*	—	(Wang, Luan, et al. 2024)
Korea	19 GC 56 (SG + IM)	Tumour tissue	GM	−70°C	Illumina MiSeq	V3–V4	—	Mann–Whitney *U* test	*—*	*Campylobacter*	—	(Kim et al. 2024)
Belgium and Lithuania	20 FD 108 GC	Tumour tissue	GM	−80°C	Illumina MiSeq	V1–V2	↓	Wilcoxon rank sum test	*Corynebacterium*	*Veillonella* *Porphyromonas* *Prevotella* *Fusobacterium* *Streptococcus*		(Oosterlinck et al. 2023)
Japan	8 EGC 9 non‐GC	Non‐specific gastric mucosa	GM	−80°C	Illumina MiSeq	V3–V4	NSD	—	—	—		(Nakano et al. 2023)
China	22 HC 22 GPL 16 GC	Not applicable	GF	0°C	Not mentioned	V4	↓	LefSe, LDA ≥ 2	*Treponema* *Streptococcus* *Lactobacillus* *Bifidobacterium* *Mobiluncus*	*Neisseria* *Treponema* *Amnipila* *Sphingomonas* *Vulcaniibacterium*, etc.	—	(Peng et al. 2023)
Lithuania	29 HC 76 GC	Tumour tissue	GM	−86°C	Illumina MiSeq	V1–V2	↓	Benjamini‐Hochberg correction	*Lactobacillus* *Clostridium sensu stricto*	*Actinomyces* *Atopobium* *Streptococcus* *Veillonella* *Prevotella*, etc.	—	(Nikitina et al. 2023)
China	30 GC 30 non‐GC	Tumour tissue	GM	−80°C	Illumina Hiseq	V4–V5	↑	LefSe, LDA > 2	*Flavonifractor* *Terrisporobacter* *Gemella* *Streptococcus* *Prevotella*, etc.	*Helicobacter* *Niveispirillum* *Acinetobacter* *Stenotrophomonas* *Escherichia‐Shigell*, etc.	—	(Nie et al. 2023)
China	60 HC 48 EGC 30 AGC	Not applicable	GF	Not mentioned	Illumina MiSeq	V1–V4	↓	LefSe, LDA > 2	*Gemella* *Lactobacillus* *Slackia*	*Chryseobacterium* *Paracoccus* *Herbaspirillum*	—	(Wei et al. 2023)
China	83 SG 21 AG 33 IM 34 FD 15 GC	Tumour tissue	GM, GF	−80°C	Not mentioned	V3–V4	↓	LefSe, LDA > 3.5	*Burkholderia‐Caballeronia‐Paraburkholderia* *Raoultella* *Enterococcus* *Streptococcus* *Lactobacillus*, etc.	*Bacteroides* *Geobacillus* *Faecalibacterium* *Blautia* *Ralstonia*, etc.	—	(Sun et al. 2022)
Singapore	17 SG 22 IM 4 EGC	Non‐specific gastric mucosa	GM	Immediate freezing	Illumina MiSeq	V3–V4	NSD	Benjamini‐Hochberg	*Enhydrobacter* *Helicobacter pylori* *Moryella* *Prevotella*	*Bifidobacterium* *Lactobacillus*	—	(Png et al. 2022)
China	30 SG 53 GC	Tumour tissue	GM	Liquid nitrogen	Ion Torrent S5 XL	V4	↓	LefSe, LDA > 4	*Oceanobacter* *Methylobacterium* *Syntrophomonas* *Prevotella* *Comamonas*, etc.	*Romboutsia* *Bifidobacterium* *Escherichia Shigella* *Neisseria* *Wolbachia*, etc.	—	(Peng et al. 2022)
China	10 GC 10 non‐GC	Tumour/lesion‐adjacent tissue	GM	−80°C	Illumina MiSeq	V3–V4	↓	LefSe, LDA > 3	*Streptococcus* *Neisseria* *Prevotella* *Veillonella* *Lactobacillus*, etc.	*Cupriavidus* *Sphingomonadales* *Sphingobium* *Brevundimonas* *Herbaspirillum*, etc.		(Shi et al. 2022)
Korea	16 SG 56 GC	Not applicable	GF	−20°C	Illumina MiSeq	V4	↓	LefSe, LDA > 4	*Lactobacillus* *Veillonella*	*Akkermansia* *Lachnospiraceae NK4A136 group*	—	(Park et al. 2022)
China	61 SG 54 IM 64 GC	Tumour and adjacent tissue	GM, GF	−80°C	Illumina Miseq	V4	↓	LefSe, LDA > 3	GM: *Lachnoanaerobaculum* *Veillonella* *Prevotella* *Fusobacterium* *Lactobacillus*, etc. GF: *Selenomonas* *Erysipelotrichaceae UCG 004* *Brevundimonas* *Lactobacillus* *Sarcina*	GM: *Helicobacter* *Carnobacterium* *Paenibacillus* GF: *Pseudomonas* *Ochrobactrum* *Methylobacterium* *Methylotenera* *Treponema 2*, etc.	—	(He, Peng, et al. 2022)
China	33 GC 36 non‐GC	Tumour tissue	GM	−80°C	Illumina NovaSeq	V4	NSD	LefSe, LDA > 4	*Prevotella*	*Klebsiella*	—	(Zhang, Hu, et al. 2022)
Korea	45 GC 92 (SG + IM)	Non‐specific gastric mucosa	GM	−70°C	Illumina Miseq	V3–V4	NSD	LefSe, LDA > 3	*Brevundimonas* *Lacticaseibacillus* *Brevundimonas vesicularis* *Lacticaseibacillus casei*	*Porphyromonas* *Schaalia* *Haemophilus* *Campylobacter* *Porphyromonas pasteri*, etc.	—	(Kim et al. 2022)
China	17 SG 10 AG 5 GPL 15 GC	Tumour/lesion‐adjacent tissue	GM	−80°C	Illumina Miseq PE300	V3–V4	NSD	LefSe, LDA > 2	*Romboutsia* *Fusicatenibacter* *Intestinimonas* *Kroppenstedtia* *Lentibacillus*, etc.	*Anaerobacillus* *Bacillus* *Massilia* *Rhodobacter* *Alloprevotella*, etc.	—	(Zhang, Li, et al. 2021)
China	25 SG 34 GC	Tumour tissue	GM	Frozen	Roche 454	Not mentioned	NSD	LefSe, LDA > 3	—	—	—	(Deng et al. 2021)
China	37 GC 37 non‐GC	Tumour tissue	GM	Not mentioned	Ion S5 TMXL	V3–V4	↑	LefSe, LDA > 3.5	*Lactobacillus* *Streptococcus* *Acinetobacter* *Prevotella* *Sphingomonas*, etc.	*Helicobacter*	—	(Dai et al. 2021)
China	21 SG 15 FGP 12 GPL 15 EGC	Tumour/lesion‐adjacent tissue	GM	Not mentioned	Illumina Miseq PE250	V3–V4	NSD	LefSe, LDA > 2	*Paracoccus* *Bacteroides* *Faecalibacterium* *Brevundimonas* *Deinococcus*, etc.	*Helicobacter* *Finegoldia* *Prevotella* *Neisseria* *Haemophilus*, etc.	—	(Li, Zhu, et al. 2021)
China	58 GC 13 non‐GC	Tumour tissue	GM	FFPE	Illumina MiSeq	V4	NSD	Student's *t* test	*Propionibacterium acnes* *Novosphingobium* *Methylobacterium fujisawaense* *Lactobacillus oris* *Comamonas aquatic*, etc.	*—*	*—*	(Li, Wu, et al. 2021)
China	30 HC 21 SG 27 IM 25 GPL 29 GC	Tumour tissue	GM	−80°C	Illumina MiSeq	V4	↓	LefSe, LDA > 3	*Helicobacter* *Lactobacillus* *Streptococcus* *Prevotella* *Veillonella*, etc.	*Halomonas* *Shewanella* *Acinetobacter* *Pseudomonas* *Aquincola*, etc.	—	(Wang, Gao, et al. 2020)
Mongolia	20 HC 20 SG 40 AG 40 IM 48 GC	Tumour tissue	GM	−80°C	Illumina MiSeq	V3–V4	↓	LefSe, LDA > 3	*Enterococcus* *Lactobacillus* *Glutamicibacter* *Paeniglutamicibacter* *Escherichia*, etc.	*Helicobacter* *Pseudomonas* *Prevotella* *Alloprevotella* *Lactobacillus*, etc.	—	(Gantuya et al. 2020)
Italy	20 GC 10 SRCC 10 ADC	Tumour tissue	GM	FFPE	Illumina MiSeq	V3–V4	NSD	LefSe, LDA > 2	SRCC: *Prevotella denticola* *Escherichia Shigella* *Fusobacterium* *Actinomyces* *Chryseobacterium*, etc. ADA: *Halomonas* *Pantoea* *Shewanella* *Lactobacillus gasseri* *Porphyromonas*, etc.	—		(Ravegnini et al. 2020)
Lithuania	22 FD 12 GC	Tumour tissue	GM	−80°C	Illumina MiSeq	V3–V4	NSD	—	—	—	—	(Spiegelhauer et al. 2020)
China	60 SG 30 EGC 30 AGC	Tumour tissue	GM	−80°C	Illumina Hiseq	V3–V4	NSD	LefSe, LDA > 2	*Ochrobactrum* *Lactobacillus* *Propionibacterium* *Serratia* *Brevibacillus*, etc.	*Deinococcus* *Devosia* *Rhizobium* *Microbacterium* *Undibacterium*, etc.	√	(Wang, Xin, et al. 2020)
Korea	288 HC 268 GC	Tumour/lesion‐adjacent tissue	GM	Not mentioned	Illumina MiSeq	V3–V4	↓	Not mentioned	*Helicobacter pylori* *Propionibacterium acnes* *Prevotella*	*Lactococcus lactis*	—	(Gunathilake et al. 2019)
China	62 GC 62 non‐GC	Tumour tissue	GM	−80°C	Illumina Hiseq 2500	V4–V5	↑	LefSe, LDA > 3	*Selenomonas* *Prevotella* *Prevotella oris* *Lachnoanaerobaculum* *Streptococcus*, etc.	*Serratia* *Helicobacter pylori* *Serratia marcescen* *Niveispirillum* *Lactococcus*, etc.		(Chen et al. 2019)
China	276 GC 276 non‐GC	Tumour and adjacent tissue	GM	Liquid nitrogen	Illumina MiSeq	V3–V4	↓	LefSe, LDA > 2	*Pseudomonas* *Enterococcus*	*Halomonas* *Shewanella*	—	(Liu, Shao, et al. 2019)
China	34 GC 34 non‐GC	Tumour tissue	GM	−80°C	Illumina MiniSeq	V4	↑	LefSe, LDA > 2	*Actinomyces* *Leptotrichia* *Neisseria* *Streptococcus* *Prevotella*, etc.	*Helicobacter*	—	(Shao et al. 2019)
China	25 GC 9 non‐GC	Tumour and adjacent tissue	GM	−80°C	Illumina MiniSeq	V4	NSD	—	—	—	—	(Liu, Vogtmann, et al. 2019)
China	59 GC 60 non‐GC	Tumour and adjacent tissue	GM	Liquid nitrogen	Illumina MiSeq	V3–V4	NSD	LefSe, LDA > 2	*Streptococcus* *Peptostreptococcus* *Lactobacillus* *Bifidobacterium* *Neisseria*, etc.	*Corynebacterium* *Cupriavidus* *Staphylococcus*		(Ling et al. 2019)
Portugal	54 SG 81 GC	Tumour and adjacent tissue	GM	−80°C	Illumina MiSeq PE300	V4	↓	LefSe, LDA > 4	*Chryseobacterium* *Phyllobacterium* *Citrobacter* *Clostridium* *Achromobacter*, etc.	*Helicobacter* *Streptococcus* *Neisseria* *Prevotella*	√	(Ferreira et al. 2018)
China	21 SG 23 AG 17 IM 20 GC	Tumour and adjacent tissue	GM	−80°C	Illumina MiSeq	V4	↓	Linear regression of R2, logistic regression	*Parvimonas micra* *Dialister pneumosintes* *Slackia exigua* *Peptostreptococcus stomatis* *Prevotella intermedia*, etc.	*Vogesella perlucida* *Acinetobacter baumannii* *Methyloversatilis discipulorum*	√	(Coker et al. 2018)
Taiwan	9 SG 7 IM 11 GC	Tumour tissue	GM	−80°C	Illumina MiSeq	V3–V4	NSD	DESeq.2 package	*Clostridium colicanis* *Fusobacterium canifelinum* *Fusobacterium nucleatum* *Lactobacillus gasseri* *Lactobacillus reuteri*, etc.	*Helicobacter pylori*	—	(Hsieh et al. 2018)
Hong Kong	8 HC 9 AG 9 IM 7 GC	Tumour and adjacent tissue	GM	−80°C	Illumina MiSeq	V3–V4	↓	LefSe, LDA > 3	*Flavobacterium* *Klebsiella* *Serratia marcescens* *Stenotrophomonas* *Achromobacter*, etc.	*Actinobacillus parahaemolyticus* *Fusobacterium* *Leptotrichia* *Helicobacter pylori* *Eubacterium*, etc.	—	(Li et al. 2017)
China	80 GC 80 non‐GC	Tumour tissue	GM	−130°C	Illumina MiSeq	V3–V4	↑	Bonferroni‐corrected	*Treponema* *Selenomonas* *Fusobacterium* *Streptococcus* *Prevotella*, etc.	*Helicobacter*	—	(Yu, Torres, et al. 2017)
Mexico	54 GC 54 non‐GC	Tumour tissue	GM	−70°C	Illumina MiSeq	V3–V4	NSD	Bonferroni‐corrected	*Clostridia*	—	—	(Yu, Torres, et al. 2017)
Malaysia and Singapore	20 FD 12 GC	Non‐specific gastric mucosa	GM	−80°C	Illumina MiSeq	V4	↑	LefSe, LDA > 2	*Rothia* *Pseudonocardia* *Bifidobacterium* *Bacteroide* *Prevotella, etc*	*Chryseobacterium* *Ruminococcus* *Bradyrhizobium* *Methylobacterium* *Sphingomonas*, etc.	—	(Castano‐Rodriguez et al. 2017)
China	80 GC 77 non‐GC	Tumour tissue	GM	−130°C	Not mentioned	Not mentioned	↓	—	—	—	—	(Yu, Hu, et al. 2017)
Korea	34 GC 29 non‐GC		GM	−80°C	GS Junior	V1–V3	NSD	—	—	—	—	(Jo et al. 2016)
Korea	1 HC 1 SG 2 GC	Non‐specific gastric mucosa	GM, GF	−80°C	GS Junior	V1–V3	NSD	—	—	—	—	(Sung et al. 2016)
China	6 SG 6 GC	Tumour/lesion‐adjacent tissue	GM	−80°C	454 GS‐FLX	V1–V3	NSD	—	—	—	—	(Wang et al. 2016)
Korea	10 AG 10 IM 11 GC	Tumour/lesion‐adjacent tissue	GM	−80°C	454 GS FLX Titanium	V5	↑	—	—	—	—	(Eun et al. 2014)
Mexico	5 SG 5 IM 5 GC	Tumour tissue	GM	−70°C	Affymetrix	V1–V9	↓	Welch test	*Lactobacillus coleohominis*	*Porphyromonas* *Neisseria* *Prevotella pallens* *Haemophilus* *Streptococcus sinensis*, etc.	—	(Aviles‐Jimenez et al. 2014)
*Prognosis*												
Lithuania	64 GC	Tumour and adjacent tissue	GM	−80°C	Illumina MiSeq	V1–V2	NSD	Log‐rank of Mantel‐Cox	*Asinibacterium*	*Fusobacterium nucleatum* *Prevotella*		(Lehr et al. 2023)
China	132 GC	Tumour/lesion‐adjacent tissue	GM	Liquid nitrogen	Illumina MiSeq	V3–V4	NSD	LefSe, LDA > 2	*Helicobacter*	*Halomonas* *Shewanella*		(Yang et al. 2023)
Belgium and Lithuania	108 GC	Tumour and adjacent tissue	GM	−80°C	Illumina MiSeq	V1–V2	—	Pearson's χ2 test	—	*Veillonella* *Porphyromonas* *Prevotella*		(Oosterlinck et al. 2023)
China	53 GC	Tumour tissue	GM	Liquid nitrogen	Ion Torrent S5 XL	V4	—	Log‐Rank Test	—	*Methylobacterium*	—	(Peng et al. 2022)
*Efficacy*												
China	48 GC	Tumour tissue	GM, FFPE	−80°C	Illumina HiSeq	Not mentioned	NSD	LefSe, LDA > 2	*Schwartzia* *Butyrivibrio*	*Slackia* *Clostridium Xivb* *Olsenella* *Megamonas* *Romboutsia*, etc.	—	(Zhang et al. 2024)

*Note:* ↑, Increased; ↓, Decreased.

Abbreviations: ADC, adenocarcinoma; AG, atrophic gastritis; AGC, advanced gastric cancer; EGC, early gastric cancer; FD, functional dyspepsia; FFPE, Formalin‐fixed, paraffin‐embedded; FGP, fundic gland polyps; GF, gastric fluid; GM, gastric mucosa; GPL, gastric precancerous lesions; *H. pylori* , *Helicobacter pylori* ; HC, healthy control; IM, intestinal metaplasia; NSD, no significant difference; RT, room temperature; SG, superficial gastritis; SRCC, signet‐ring cell carcinoma.

Although most reports have explored a notable decrease in microbial diversity in GC groups compared to controls (Aviles‐Jimenez et al. 2014; Li et al. 2017; Yu, Hu, et al. 2017; Coker et al. 2018; Ferreira et al. 2018; Gunathilake et al. 2019; Liu, Shao, et al. 2019; Gantuya et al. 2020; Wang, Gao, et al. 2020; He, Peng, et al. 2022; Park et al. 2022; Peng et al. 2022, 2023; Shi et al. 2022; Sun et al. 2022; Nikitina et al. 2023; Oosterlinck et al. 2023; Wei et al. 2023), other studies have observed either an increase in microbial diversity (Eun et al. 2014; Castano‐Rodriguez et al. 2017; Yu, Torres, et al. 2017; Chen et al. 2019; Shao et al. 2019; Dai et al. 2021; Nie et al. 2023; Lei et al. 2024; Qian et al. 2024) or no significant differences between GC and control groups (Jo et al. 2016; Sung et al. 2016; Wang et al. 2016; Yu, Torres, et al. 2017; Hsieh et al. 2018; Ling et al. 2019; Liu, Vogtmann, et al. 2019; Ravegnini et al. 2020; Spiegelhauer et al. 2020; Wang, Xin, et al. 2020; Deng et al. 2021; Li, Zhu, et al. 2021; Li, Wu, et al. 2021; Zhang, Li, et al. 2021; Kim et al. 2022; Png et al. 2022; Zhang, Hu, et al. 2022; Nakano et al. 2023). One study compared the microbiota composition between two GC subtypes—signet‐ring cell carcinoma and adenocarcinoma—and found no significant differences in microbial diversity (Ravegnini et al. 2020). Furthermore, studies have shown no correlation between gastric microbiota diversity and GC prognosis or treatment efficacy (Lehr et al. 2023; Yang et al. 2023; Zhang et al. 2024).

In addition to microbial diversity, current studies have identified significant differences in microbial composition associated with GC patients, as well as their varying prognoses and treatment outcomes (Table 1). Briefly, some research suggested that *Lactobacillus*, *Prevotella*, *Streptococcus*, and *Fusobacterium* were significantly enriched in GC groups, while *Helicobacter*, *Bifidobacterium*, *Neisseria*, and *Acinetobacter* were remarkably depleted (Yu, Torres, et al. 2017; Ferreira et al. 2018; Dai et al. 2021; Peng et al. 2022; Qian et al. 2024). Moreover, the high abundance of *F. nucleatum* , *Prevotella*, *Halomonas*, *Shewanella*, *Methylobacterium, Neisseria*, and *Veillonella* was significantly associated with poor prognosis, whereas *Asinibacterium* and *H. pylori* were more abundant in GC patients with good prognosis (Peng et al. 2022; Lehr et al. 2023; Oosterlinck et al. 2023; Yang et al. 2023). Notably, *Planococcaceae, Rhizobiales*, and *Carnobacteriaceae* were obviously enriched in patients responsive to neoadjuvant therapy, while *Rhodococcus, Caulobacter*, and *Negativicutes* were enriched in non‐responders (Zhang et al. 2024).

The above research reveals that GC patients do have a unique microbial composition and diversity, but the differentially abundant taxonomic features are not identical across studies. These heterogeneities are likely attributable to variations in study design and execution (Table 1). For instance, studies performed across different regions exhibit marked differences in factors such as sample size, control group selection, sample source (e.g., GM vs. GF), and tissue type of GC cases (e.g., tumour tissue versus tumour/lesion‐adjacent tissue). The gastric microbiota exhibits distinct characteristics in tumour and tumour/lesion‐adjacent tissues. Tumour tissues typically show reduced microbial diversity due to hypoxic and inflammatory conditions that favour the growth of specific anaerobic bacteria, such as *Fusobacterium*. In contrast, tumour/lesion‐adjacent tissues, while influenced by the tumour microenvironment, may retain a more diverse microbiota that represents an intermediate state between healthy and malignant tissues. These disparities are further amplified by differences in sequencing platforms, targeted sequencing regions, and the analysis method of 16S rRNA data. Such methodological and procedural variations hinder the comparability of results. Resolving the above heterogeneities is essential for enhancing the reliability and generalisability of future research findings.

Given the strong association between *H. pylori* infection and GC, several studies have investigated changes in gastric microbiota composition with or without *H. pylori* infection. Numerous studies have demonstrated that the microbial diversity in *H. pylori* ‐negative individuals is significantly higher than in *H. pylori* ‐positive individuals, and successful eradication of *H. pylori* can partially recover the gastric microbiota (Li et al. 2017, 2023; Chen et al. 2019; Gantuya et al. 2019, 2020; He et al. 2019; Liu, Shao, et al. 2019; Guo et al. 2020; Miao et al. 2020; Spiegelhauer et al. 2020; Sung et al. 2020; Wang, Gao, et al. 2020; Mao et al. 2021; Watanabe et al. 2021; He, Peng, et al. 2022; Liu, Wang, and Xie 2022; Wang, Luan, et al. 2024). In GC patients without *H. pylori* infection, increased microbial abundance was associated with significantly improved survival and chemotherapy outcomes (Lei et al. 2024). On the other hand, *H. pylori* infection distinctly alters the composition of the gastric microbiota. In *H. pylori* ‐positive samples, the abundance of *Serratia*, *Lactobacillus*, and *Streptococcus* is higher, whereas in the *H. pylori* ‐negative group, *Pseudomonas aeruginosa* and its higher taxonomic levels from genus to phylum are significantly enriched (Chen et al. 2019). Furthermore, by eradicating *H. pylori* , the abundance of *Cyanobacteria*, *Bacteroidetes*, *Fusobacteria*, *Actinobacteria*, and *Firmicutes* increases significantly (Guo et al. 2020). In the GC microenvironment, *Prevotella* is predominantly enriched in areas without *H. pylori* infection, whereas *Oribacterium* is more prevalent in areas with *H. pylori* infection (Wang, Luan, et al. 2024). These results indicate that *H. pylori* infection profoundly affects the microecological balance through competitive and ecological mechanisms. *H. pylori* eradication therapy not only alleviates dysbiosis but may also provide a protective role in the prevention and management of gastric diseases. Future studies should focus on combining microbiota remodelling strategies to optimise treatment outcomes.

### 2.2 16S rRNA‐Based Microbial Diversity and Composition of GC From Oral Samples

The oral microbiota plays a crucial role in shaping the composition of the gastrointestinal microbiota. Table 2 summarises current findings on the microbial diversity and composition of oral samples.

**TABLE 2:** The studies on microbiota characteristics in oral samples from GC patients based on 16S rRNA gene sequencing.

Country	Sample size	Sample source	Storage condition	Sequencing platform	Sequencing region	Diversity	Taxonomic discovery analysis	Significantly enriched in GC patients (genus and species level)	Significantly depleted in controls (genus and species level)	External validation	Ref
*Diagnosis*											
China	70 HC 70 GC	Oral swab samples	−80°C	Illumina NovaSeq	V4	↓	LefSe, LDA > 4	*Leptotrichia* *Gemella*	*Haemophilus* *Haemophilus parainfluenzae* *Neisseria* *Neisseria mucosa*	—	(Zhang, Hu, et al. 2022)
China	34 HC 33 GC	Saliva	−20°C	Ion S5TM XL	V3–V4	NSD	Wilcoxon test	*Alloprevotella* *Prevotella* *Megasphaera* *Megasphaera micronuciformis* *Leptotrichia*, etc.	*Bergeyella* *Granulicatella* *Streptococcus salivarius* *TM7 G‐6*	—	(Shu et al. 2022)
China	101 SG 93 AG 99 GC	Saliva	RT	Illumina MiSeq PE300	V3–V4	↓	LefSe, LDA > 3.4	*Streptococcus* *Bifidobacterium* *Unclassified Streptophyta*	*Anaerovorax* *Bulleidia* *Fusobacterium* *Neisseria* *Parvimonas*, etc.	—	(Huang et al. 2021)
China	112 HC 181 GC	Tongue coating	−80°C	Illumina MiSeq	V3–V4	↓	LefSe, LDA > 3	*Lactococcus* *Megamonas* *Geobacillus* *Pseudomonas* *Faecalibacterium*	*Peptococcus* *Rothia* *Haemophilus* *Neisseria*, etc.	—	(Xu et al. 2021)
Japan	118 HC 10 GC	Saliva	−80°C	Ion PGM	V1–V2	NSD	LefSe, LDA > 3	*Haemophilus parainfluenzae* *Fusobacterium periodonticum* *Gemella sanguinis* *Streptococcus* *Neisseria*	*Rothia* *Streptococcus* *Lachnospiraceae (G‐2) sp. (096)* *Prevotella* *Porphyromonas*	—	(Kageyama et al. 2019)
China	80 HC 57 GC	Tongue coating	−80°C	Roche Genome Sequencer FLX+ Instrument	V4	NSD	Benjamini‐Hochberg	*Abiotrophia* *Corynebacterium* *Lactobacillus* *Anaeroglobus* *Streptococcus*, etc.	*Neisseria* *Prevotella* *Porphyromonas* *Lachnoanaerobaculum* *Haemophilus*, etc.	—	(Wu et al. 2018)
China	13 HC 37 GC	Saliva and plaque	−70°C	Illumina MiSeq	V4	↑	Not mentioned	*Veillonella* *Prevotella* *Capnocytophaga* *Aggregatibacter* *Megasphaera*	*Campylobacter* *Tannerella* *Granulicatella* *Leptotrichia* *Rothia*	—	(Sun et al. 2018)
China	72 HC 74 GC	Tongue coating	−80°C	Illumina MiSeq	V2–V4	↓	Student–Newman–Keuls	*Prevotella* *Streptococcus* *Veillonella* *Actinomyces* *Leptotrichia*	*Fusobacterium* *Neisseria* *Haemophilus* *Porphyromonas*		(Hu et al. 2015)

*Note:* ↑, Increased; ↓, Decreased.

Abbreviations: AG, atrophic gastritis; GC, gastric cancer; HC, healthy control; NSD, no significant difference; RT, room temperature; SG, superficial gastritis.

Some reported reduced oral microbiota diversity in GC patients compared to healthy controls (Hu et al. 2015; Huang et al. 2021; Xu et al. 2021; Zhang, Hu, et al. 2022). However, some found no significant differences in oral microbiota diversity between GC patients and controls (Wu et al. 2018; Kageyama et al. 2019; Shu et al. 2022), while a particular study observed greater complexity in the oral microbiota of GC patients (Sun et al. 2018).

In the study of the complexity of oral microbiota and its association with GC, *H. pylori* can be detected in dental plaque, saliva, tongue coating, and dental pulp (Mendoza‐Cantu et al. 2017; Nomura et al. 2018; Zhao et al. 2019; Ji et al. 2020). Amongst these, the detection of *H. pylori* in saliva is particularly significant, as saliva may act as a medium for bacterial transmission and reinfection. However, there are currently no reports on the differences in saliva microbiota amongst different *H. pylori* infection statuses. This oral reservoir of *H. pylori* can aggravate the severity of gastric infection and increase the difficulty of eradication, which may serve as a major source of reinfection (Miyabayashi et al. 2000; Ansari et al. 2018).

Furthermore, studies have revealed that the oral microbiota of GC patients exhibit enriched *Leptotrichia*, *Veillonella*, and *Streptococcus*, but reduced *Haemophilus*, *Neisseria*, and *Prevotella* (Hu et al. 2015; Kageyama et al. 2019; Zhang, Hu, et al. 2022). Notably, in non‐ *H. pylori* ‐infected GC, infecting certain oral microbes, such as *V. parvula* and *S. oralis* , was associated with poor survival outcomes and chemotherapy efficacy (Lei et al. 2024).

Similar to stomach samples, the relationship between oral microbial diversity or composition and GC remains inconsistent across studies (Table 2). These heterogeneities may also be attributed to variations in sample sizes, control groups, and sample types, such as tongue coating and saliva. Overall, more rigorous and standardised study designs are urgently needed to enhance the reliability and comparability of future research.

### 2.3 16S rRNA‐Based Microbial Diversity and Composition of GC From Stool Samples

Stool microbiota biomarkers have shown significant potential for early screening of colorectal cancer, while the application in GC screening is still under discussion. Studies analysing microbial DNA from stool samples have revealed a strong association between the composition of gut microbiota and the development of GC. Table 3 provides a summary of current findings on microbial diversity and composition of stool samples associated with GC.

**TABLE 3:** The studies on microbiota characteristics in stool samples from GC patients based on 16S rRNA gene sequencing.

Country	Sample size	Storage condition	Sequencing platform	Sequencing region	Diversity	Taxonomic discovery analysis	Significantly enriched in GC patients/good prognosis patients/no response patients (genus and species level)	Significantly depleted in controls/poor prognosis patients/response patients (genus and species level)	External validation	Ref
*Diagnosis*										
China	35 EGC 32 AGC	−80°C	Illumina NovaSeq	V4	NSD	LefSe	AGC: *Collinsella* *Blautia* *Anaerostipes* *Dorea* *Lachnospiraceae ND3007 group*	—	—	(Chen, Du, et al. 2022)
China	468 SG 43 GPL 532 GC	Not mentioned	Illumina MiSeq	V3‐V4	—	Multiple hypothesis tests	*Streptococcus anginosus* *Streptococcus constellatus*	—	√	(Zhou et al. 2022)
China	147 HC 46 GC	−80°C	Illumina HiSeq2500	V4	NSD	LefSe, LDA ≥ 3.6	*Fusobacterium* *Clostridium* *Parabacteroides* *Escherichia* *Akkermansia*, etc.	*Clostridia* *Faecalibacterium* *Roseburia* *Lachnospira* *Bifidobacterium*, etc.	—	(Li et al. 2022)
China	30 HC 30 GC	−80°C	Illumina NovaSeq	V4	NSD	LefSe, LDA ≥ 2.0	*Streptococcus* *Enterococcus* *Lactobacillus* *Blautia* *Romboutsia*, etc.	*Megamonas* *Roseburia* *Subdoligranulum* *Agathobacter* *Faecalibacterium*	—	(He, Wang, et al. 2022)
China	30 HC 22 GC	−80°C	Illumina MiSeq	V3–V4	NSD	LefSe, LDA ≥ 2.0	*Streptococcus* *Ruminococcus gnavus group* *Candidatus Nitrotoga* *Hungatella* *Erysipelatoclostridium*, etc.	*Mucilaginibacter* *Chujaibacter* *Phenylobacterium* *Granulicella* *Pandoraea*, etc.		(Zhang, Zhu, et al. 2022)
China	70 HC 49 GC	−80°C	Illumina NovaSeq	V4	↑	LefSe, LDA > 4	*Escherichia–Shigella* *Escherichia coli* *Dialister* *Streptococcus*	*Faecalibacterium* *Faecalibacterium prausnitzii*	—	(Zhang, Hu, et al. 2022)
China	15 SG 13 AG 8 GPL 15 GC	−20°C	Illumina NovaSeq	V4	↓	LefSe, LDA > 3	*Lachnospiraceae unclassified* *Tyzzerella 3* *Actinomyces* *Burkholderiales unclassified* *Peptoniphilus*, etc.	*Dorea* *Faecalibacterium* *Roseburia* *Lachnoclostridium* *Fusicatenibacter*, etc.	—	(Miao et al. 2022)
China	147 HC 46 GC	−80°C	Illumina HiSeq2500	V4	NSD	LefSe, LDA ≥ 3.6	GIC: *Bacteroides fragilis* *Escherichia coli* *Akkermansia muciniphila* *Clostridium hathewayi* *Alistipes finegoldii*	*Faecalibacterium*prausnitzii *Roseburia faecis* *Clostridium clostridioforme* *Blautia producta* *Bifidobacterium adolescent*, etc.		(Li, Bai, et al. 2021)
China	61 HC 54 AG 83 GC	−80°C	Illumina HiSeq4000	V4	↑	LefSe, LDA > 3	*Veillonella* *Streptococcus* *Akkermansia* *Haemophilus* *Actinomyces*, etc.	*Paraprevotella* *Acinetobacter* *Ruminococcus*	—	(Zhang, Shen, et al. 2021)
China	49 HC 49 GC	−80°C	Illumina	V3‐V4	NSD	LefSe, LDA > 4	*Lactobacillus* *Streptococcus* *Fusobacterium*	*Roseburia* *Blautia*	—	(Yu et al. 2021)
China	35 HC 38 GC	−80°C	454GS‐FLX system	V3‐V4	↓	Student's t‐tests	*Proteobacteria* *Escherichia* *Desulfovibrio*	*Faecalibacterium* *Roseburia* *Lachnospira* *Anaerostipes*	—	(Liu et al. 2021)
Finland	13 HC 29 GC 13 DGC 15 IGC 2 MGC	Not mentioned	Ion PGM	V2, V3, V4, V6‐7, V8, V9	↓	ALDEx2	—	*Oscillibacter* *Lachnoclostridium* *Bifidobacterium* *Parabacteroides* *Barnesiella*		(Sarhadi et al. 2021)
China	58 HC 134 GC	−80°C	Illumina Hiseq	V3–V5	NSD	Pearson correlation	*Veillonella* *Streptococcus mitis* *Megasphaera* *Streptococcus salivarius* *Bifidobacterium dentium*, etc.	—	—	(Wu et al. 2020)
China	88 HC 116 GC	−80°C	Illumina MiSeq	V3–V4	NSD	LefSe, LDA > 3.5	*Prevotella 9* *Escherichia– Shigella* *Klebsiella* *Lactobacillus* *Streptococcus*, etc.	*Lachnoclostridium* *Eubacterium rectale* *Roseburia* *Lachnospira* *Faecalibacterium*	—	(Qi et al. 2019)
China	22 HC 20 GC	−80°C	Illumina MiSeq	Not mentioned	↓	—	—	—	—	(Liang et al. 2019)
China	38 PM 54 non‐PM	−80°C	Illumina	V3–V4	NSD	LefSe, LDA > 2	*Roseburia* *Ruminococcus* *Coprococcus* *Collinsella* *Dorea*, etc.	*Veillonella* *Subdoligranulum* *Cloacibacillus* *Psychrobacter* *Sphingobacterium*, etc.	√	(Yu et al. 2024)
*Prognosis*										
China	49 CT 107 non‐CT	−80°C	Illumina HiSeq	V4	NSD	*t* Test	Ki67(−) and HER2(−): *Blautia* *Ruminococcus* *Oscillospira* *Lachnospira*	Ki67(+) and HER2(+): *Prevotella* *Eubacterium* *Desulfovibrio*	—	(Chen, Shen et al. 2022)
China	13 LM 13 non‐LM	−80°C	Illumina	V3‐V4	NSD	LefSe, LDA > 3	*Macellibacteroides*	*Streptococcus* *Gemmiger* *Butyricicoccus*	—	(Yu et al. 2021)
*Efficacy*										
China	46 GC	−80°C	Illumina HiSeq2500	V4	NSD	—	—	—	—	(Li, Bai, et al. 2021)
China	89 GIC	RT	Illumina HiSeq2500	V3–V4	NSD	Wilcoxon test	*Megamonas* *Butyricimonas* *Lachnospiraceae_UCG‐001* *Agathobacter*	*Prevotella* *Bifidobacterium* *Bacteroides*	—	(Peng et al. 2020)

*Note:* ↑, Increased; ↓, Decreased.

Abbreviations: AG, atrophic gastritis; AGC, advanced gastric cancer; CT, chemotherapy; DGC, diffuse gastric cancer; EGC, early gastric cancer; GC, gastric cancer; GIC, gastrointestinal cancer; GPL, gastric precancerous lesions; HC, healthy control; IGC, intestinal gastric cancer; LM, liver metastasis; MGC, mixed gastric cancer; NSD, no significant difference; PM, peritoneal metastasis; RT, room temperature; SG, superficial gastritis.

Many studies suggested that there was no significant change in stool microbiota diversity in GC patients compared to controls (Qi et al. 2019; Wu et al. 2020; Li, Bai, et al. 2021; Yu et al. 2021; Chen, Du, et al. 2022; He, Wang, et al. 2022; Li et al. 2022; Zhang, Zhu, et al. 2022). Conversely, some studies reported reduced diversity (Liang et al. 2019; Liu et al. 2021; Miao et al. 2022), while others found an increase in stool microbiota diversity of GC relative to that of healthy individuals (Zhang, Shen, et al. 2021; Zhang, Hu, et al. 2022). Studies on different GC subtypes have shown that patients with diffuse adenocarcinoma exhibit lower gut microbiota diversity compared to those with intestinal adenocarcinoma (Sarhadi et al. 2021). Further investigations indicated no significant differences in gut microbial diversity between GC patients with and without liver or peritoneal metastases, before and after chemotherapy, or between responders and non‐responders to immune checkpoint inhibitors (Peng et al. 2020; Li, Bai, et al. 2021; Yu et al. 2021, 2024; Chen, Shen, et al. 2022).

The compositional changes in gut microbiota are likely to be associated with the progression of GC. In GC patients, there is a notable depletion of beneficial bacteria such as *Faecalibacterium, Roseburia, Lachnospira*, and *Bifidobacterium*, alongside an enrichment of pathogenic bacteria, including *Desulfovibrio, Streptococcus, Fusobacterium*, and *Escherichia* (Liu et al. 2021; He, Wang, et al. 2022; Li et al. 2022; Zhou et al. 2022). Additionally, different gastric tumour subtypes (e.g., diffuse and intestinal adenocarcinoma) have been associated with a high abundance of *Enterobacteriaceae*, while *Bifidobacteriaceae* is less abundant only in diffuse adenocarcinoma, and *Oscillibacter* is less abundant in intestinal adenocarcinoma (Sarhadi et al. 2021). Amongst GC patients' responses to immune checkpoint inhibitors, significant enrichment of *Prevotella, Bifidobacterium, Bacteroides, Ruminococcaceae*, and *Lachnospiraceae* is observed (Peng et al. 2020), while non‐responders exhibit higher abundances of *Megamonas, Butyricimonas, Lachnospiraceae_UCG‐001*, and *Agathobacter* (Peng et al. 2020).

Similarly to stomach or oral samples, variations in regions, sample types, sample sizes, sequencing platforms, and data analysis methods have contributed to heterogeneous conclusions on the richness and diversity of microbiota in stool samples from GC patients (Table 3).

## 3 16S rRNA‐Based Microbial Biomarkers for GC Diagnosis and Prognosis

The present studies underscore the potential of microbiota profiles derived from stomach, oral, and stool samples to effectively distinguish between GC and non‐GC patients. Recent research has also focused on identifying 16S rRNA‐based microbial biomarkers for GC diagnosis and prognosis. It is worth noting that specific microbiota alterations emerge at different stages of cancer and may serve as specific biomarkers with greater diagnostic precision compared to conventional methods. Moreover, using non‐invasive samples, such as saliva or stool, seems to enhance patient compliance and improve the practicality of GC screening.

### 3.1 16S rRNA‐Based Microbial Biomarkers for GC Diagnosis

To date, a total of 24 studies and 4 meta‐analyses have explored the value of microbial biomarkers in GC diagnosis (Table 4). Amongst 12 studies focusing on stomach samples, the diagnostic performance of microbial biomarkers, as indicated by the area under the curve (AUC), ranged from 0.77 to 1.0. For instance, Wang, Gao, et al. (2020) identified a set of 5 microbial biomarkers that distinguished GC patients from non‐GC individuals with an AUC of 0.99. Similarly, He, Peng, et al. (2022) differentiated GC from superficial gastritis (SG) using 7 GM and 13 GF biomarkers, achieving AUCs of 0.84 and 0.89, respectively. Another study utilised He, Peng, et al.'s (2022) dataset to successfully distinguish GC from non‐GC samples, reporting an AUC of 0.86 for GM and 0.71 for GF microbiota (Li et al. 2023). Notably, after excluding *H. pylori* sequences, the AUC improved to 0.75 for GF samples and 0.91 for GM samples, underscoring the significant impact of *H. pylori* on microbial composition and its pivotal role in GC progression (Li et al. 2023). Amongst the gastric microbial markers, *Lactobacillus*, *Helicobacter*, and *Streptococcus* appeared more frequently, suggesting that they may be directly involved in gastric mucosa carcinogenesis. As stomach samples are closest to the lesion site, they provide highly specific microbiota data and enable detailed investigations into the interactions between *H. pylori* and other microbial communities. However, the invasive nature of biopsy procedures may limit patient acceptance, and the presence of *H. pylori* could obscure other key microbial features. Moreover, certain microbial markers may exhibit varying diagnostic value depending on whether they are derived from tumour tissue or tumour/lesion‐adjacent tissue. For instance, *Fusobacterium* may demonstrate robust diagnostic potential in tumour tissue but be less relevant in tumour/lesion‐adjacent tissue. Similarly, *H. pylori* is more prevalent in paracancerous tissues and may serve as a more reliable indicator of early GC. Therefore, although current models based on 16S rRNA microbial biomarkers demonstrate remarkable diagnostic performance for GC, their generalisability across diverse populations requires further validation.

**TABLE 4:** Overview of 16S rRNA‐based microbial biomarkers for GC diagnosis and prognosis.

Biomarkers	Sample	Tissue type of GC cases	AUC	Ref
*Diagnosis*				
15 biomarkers (*Streptococcus, Lactobacillus, Ochrobactrum, Rhodococcus, JG30‐KF‐CM45, Paracoccus, Capnocytophaga, Gemella, Rothia, Treponema, Acinetobacter, Stenotrophomonas, Corynebacterium, Atopobium, Helicobacter*)	GF	Not applicable	1.00	(Wei et al. 2023)
*Lacticaseibacillus*, *Haemophilus*, *Campylobacter*, *TWIST1* methylation, age, sex, atrophy	GM	Tumour tissue	0.994	(Kim et al. 2024)
5 biomarkers (*Sphingobium, Lactobacillus, Aquincola tertiaricarbonis, Bacillus, Acinetobacter johnsonii*)	GM	Tumour tissue	0.99	(Wang, Gao, et al. 2020)
24 biomarkers (*Pseudonocardia, Halomonas, Pseudoxanthomonas, Arthrobacter, Ralstonia, Anoxybacillus, Gordonia, Undibacterium, Rhizobium, Novosphingobium, Burkholderia, Uruburuella, Paenibacillus, Aquabacterium, Sphingomonas, Methylobacterium, Ochrobactrum, Bradyrhizobium, Salinivibrio, Tsukamurella, Pelomonas, Enhydrobacter, Paracoccus, Dietzia*)	GM	Tumour tissue	0.97 (EGC vs. SG)	(Wang, Xin, et al. 2020)
			0.84 (EGC vs. AGC)	
5 biomarkers ( *Clostridium colicanis* , *Fusobacterium canifelinum* , *Fusobacterium nucleatum* , *Lactobacillus gasseri* , *Lactobacillus reuteri* )	GM	Tumour tissue	0.938	(Hsieh et al. 2018)
*Syntrophomonas*	GM	Tumour tissue	0.9352	(Peng et al. 2022)
*Oceanobacter*			0.9062	
*Methylobacterium*			0.7732	
10 most relevant taxa (the microbial dysbiosis index)	GM	Tumour and adjacent tissue	0.89	(Ferreira et al. 2018)
13 biomarkers (*Ochrobactrum*, *Lactobacillus*, *Eubacterium nodatum group*, *Methylobacterium*, *Filifactor*, *Selenomonas*, *Staphylococcus*, *Treponema 2*, *Peptococcus*, *Capnocytophaga*, *Leptotrichia*, *Dialister*, *Rikenellaceae RC9 gut group*)	GF	Not applicable	0.89	(He, Peng, et al. 2022)
7 biomarkers (*Lactobacillus*, *Enhydrobacter*, *Veillonella*, *Gemella*, *Enterococcus*, *Helicobacter*, *Paenibacillus*)	GM	Tumour and adjacent tissue	0.84	
6 biomarkers (*Moryella, Vibrio, Comamonadaceae, Paludibacter, Agrobacterium, Clostridiales*)	GM	Non‐specific gastric mucosa	0.82	(Png et al. 2022)
5 biomarkers ( *Peptostreptococcus stomatis* , *Streptococcus anginosus* , *Parvimonas micra* , *Slackia exigua* , *Dialister pneumosintes* )	GM	Tumour and adjacent tissue	0.81	(Coker et al. 2018)
*Fusobacterium*	GM	Tumour and adjacent tissue	0.836	(Wang, Luan, et al. 2024)
*Lactobacillus*			0.770	
*Leptotrichia*			0.882	
*Prevotella*			0.822	
*Streptococcus*			0.937	
*Veillonella*			0.780	
*Campylobacter*			0.942	
*Helicobacter*			0.884	
*Oribacterium*			0.841	
*Rothia*			0.955	
4 biomarkers ( *Helicobacter pylori* , *Propionibacterium acnes* , *Prevotella*, *Lactococcus lactis* )	GM	Tumour/lesion‐adjacent tissue	0.777	(Gunathilake et al. 2019)
10 biomarkers (*Veillonella*, *Prevotella*, *Capnocytophaga*, *Aggregatibacter*, *Megasphaera*, *Campylobacter*, *Tannerella*, *Granulicatella*, *Leptotrichia*, *Rothia*)	Saliva and plaque	Not applicable	0.97 (sensitivity based on GC screening score system)	(Sun et al. 2018)
10 biomarkers (*Unclassified Streptophyta*, *Haemophilus*, *Streptococcus*, *Unclassified Mogibacteriaceae*, *Peptostreptococcus*, *Corynebacterium*, *Dialister*, *Unclassified TM73*, *Neisseria*, *Lautropia*)	Saliva	Not applicable	0.91	(Huang et al. 2021)
6 biomarkers (*Peptostreptococcus*, *Peptococcus*, *Porphyromonas*, *Megamonas*, *Rothia*, *Fusobacterium*)	Tongue coating	Not applicable	0.85	(Xu et al. 2021)
10 biomarkers (*Atopobium*, *Ruminococcaceae UCG‐014*, *Stomatobaculum*, *Candidatus Saccharimonas*, *Lachnospiraceae uncultured*, *Oribacterium*, *Prevotella 7*, *Eubacteriumnodatumgroup*, *Prevotella*, *Erysipelotrichaceae UCG‐007*)	Tongue coating	Not applicable	0.76	(Wu et al. 2018)
9 OTU markers	Stool	Not applicable	0.9711	(Li et al. 2022)
5 biomarkers (*Lactobacillus*, *Tyzzerella 3*, *Veillonella*, *Streptococcus*, *Lachnospira*)	Stool	Not applicable	0.95	(Qi et al. 2019)
4 biomarkers (*Desulfovibrio*, *Escherichia*, *Faecalibacterium*, *Oscillospira*)	Stool	Not applicable	0.907	(Liu et al. 2021)
2 biomarkers ( *Streptococcus anginosus* , *Streptococcus constellatus* )	Stool	Not applicable	0.87	(Zhou et al. 2022)
*Veillonella*	Stool	Not applicable	0.85	(Wu et al. 2020)
*Streptococcus ASV‐63689*	Stool	Not applicable	0.842	(Yu et al. 2021)
*Streptococcus ASV‐61493*			0.772	
28 biomarkers (*Lactobacillus*, *Megasphaera*, etc.)	Stool		0.83	(Zhang, Shen, et al. 2021)
13 oral biomarkers (*Alloprevotella*, *Prevotella*, *F0332*, *Streptococcus anginosus* , *Kingella*, *Prevotella pallens* , *Eikenella corrodens* , *Ottowia sp. oral taxon 894*, *Firmicutes oral clone BB124*, *Mitsuokella sp. oral taxon G68*, *Veillonella*, *Neisseria*, *Lautropia*) 9 faecal biomarkers (*Lachnospira*, *Clostridium perfringens* , *Streptococcus anginosus* , *Lactobacillus*, *Lactobacillus mucosae* , *Parasutterella secunda* , *Peptococcus*, *Paraprevotella*, *Streptococcus*)	Oral	Not applicable	0.824	(Zhang, Hu, et al. 2022)
	Stool		0.939	
	Oral and Stool	Not applicable	0.922	
8 biomarkers (*Veillonella*, *Dialister*, *Granulicatella*, *Herbaspirillum*, *Comamonas*, *Chryseobacterium*, *Shewanella*, *Helicobacter*)	Met‐analysis	Not applicable	0.85	(Liu, Ng, et al. 2022)
17 biomarkers (*Helicobacter*, *Acinetobacter*, *Streptococcus*, *Sphingomonas*, *Haemophilus*, *Halomonas*, *Vibrio*, *Pseudomonas*, *Veillonella*, *Selenomonas*, *Gilliamella*, *Peptostreptococcus*, *Snodgrassella*, *Leptotrichia*, *Hydrogenophilus*, *Bacillus*, *Bifidobacterium*)	Meta‐analysis	Not applicable	0.908 (GC vs. SG)	(Liu, Zhang, et al. 2022)
22 biomarkers (*Acinetobacter*, *Peptostreptococcus*, *Helicobacter*, *Lactobacillus*, *Pseudomonas*, *Haemophilus*, *Porphyromonas*, *Neisseria*, *Selenomonas*, *Vibrio*, *Campylobacter*, *Shewanella*, *Gilliamella*, *Actinobacillus*, *Sphingomonas*, *Lachnospiraceae NK4A136 group*, *Hydrogenophilus*, *Turicibacter*, *Brevundimonas*, *Kocuria*, *Rhodococcus*, *Sarcina*)			0.964 (GC vs. GPL)	
5 biomarkers (*Streptococcus*, *Peptostreptococcus*, *Selenomonas*, *Pseudomonas*, *Prevotella*)	Meta‐analysis		0.7525 (Matched)	(Chen et al. 2023)
			0.8818 (Unmatched)	
			0.7435 (Others)	
2 biomarkers (*Lactobacillus*, *Streptococcus*)	Meta‐analysis		0.7712	(Wang, Wang, et al. 2024)
*Prognosis*				
OS: 3 biomarkers (*Helicobacter*, *Halomonas*, *Shewanella*)	GM	Tumour/lesion‐adjacent tissue	0.749	(Yang et al. 2023)
LM: *Gemmiger*	Stool	Not applicable	0.751	(Yu et al. 2021)
*Streptococcus*			0.651	
*Bacteroides*			0.751	
PM: *Roseburia*	Stool	Not applicable	0.698	(Yu et al. 2024)

Abbreviations: AGC, advanced gastric cancer; AUC, area under the curve; EGC, early gastric cancer; GF, gastric fluid; GM, gastric mucosa; GPL, gastric precancerous lesions; LM, liver metastasis; OS, overall survival; PM, peritoneal metastasis; SG, superficial gastritis; TWIST1, twist family bHLH transcription factor 1.

Regarding oral samples, five studies have reported the potential value of microbial biomarkers in GC diagnosis, with diagnostic performance ranging from 0.76 to 0.97. Notably, one study developed a GC screening score based on 10 oral biomarkers, achieving an impressive sensitivity of 97% (Sun et al. 2018). Amongst the commonly identified microorganisms in these studies are *Peptostreptococcus*, *Prevotella*, *and Rothia*. Although oral microbiota do not directly interact with the stomach, they may contribute to GC development through mechanisms such as gastric microecology, inducing chronic inflammation, or producing carcinogenic metabolites. Oral‐derived samples offer several advantages: they are easy to collect, non‐invasive, and highly suitable for large‐scale screening. Moreover, they may reflect microbial changes associated with the early stages of GC, offering potential as early diagnostic tools. However, the specificity of oral microbes as biomarkers can be affected by external factors such as diet and oral hygiene, which may influence the microbial composition. Therefore, further research is needed to enhance their diagnostic precision and reliability, particularly in standardising sampling methods and accounting for confounding factors.

With respect to stool samples, eight studies have reported on the value of microbial biomarkers in GC diagnosis, with diagnostic performance ranging from 0.772 to 0.9711. A predictive model based on nine operational taxonomic units (OTUs) of gut microbiota achieved an impressive AUC of 0.9711, specifically for the GC population (Li et al. 2022). Furthermore, a large multi‐centre study identified *S. anginosus* and *S. constellatus* as potential diagnostic biomarkers for early‐stage GC, demonstrating an AUC of 0.87 (Zhou et al. 2022). Amongst stool microbiota reported as potential diagnostic biomarkers for GC, *Streptococcus*, *Veillonella*, and *Lactobacillus* have been most frequently studied. Notably, *Streptococcus* and *Lactobacillus* also appear as oral microbiota biomarkers, suggesting their potential involvement in GC pathogenesis, which merits further exploration. Stool samples offer several advantages, including ease of collection, non‐invasiveness, and high patient compliance, making them ideal for large‐scale, multi‐centre studies. However, the inherent complexity of the gut microbiota and the diverse intestinal microbial background may dilute or obscure GC‐specific biomarkers. Additionally, the indirect relationship between gut microbiota and gastric microbiota may limit their utility in the precise diagnosis of GC, highlighting the need for further investigation.

Additionally, other studies have explored the use of various combinations of 16S rRNA data or the integration of additional patient information to identify diagnostic biomarkers for GC. For instance, a diagnostic model combining oral and faecal biomarkers achieved promising results, with an AUC of 0.922 for GC, which is better than the efficiency of models based solely on oral microbiota markers (AUC = 0.824) (Zhang, Hu, et al. 2022). Moreover, an advanced predictive model combining microbial signatures of *Lacticaseibacillus*, *Haemophilus*, and *Campylobacter* with twist family bHLH transcription factor 1 methylation levels, age, sex, atrophic gastritis, and intestinal metaplasia demonstrated exceptional accuracy in identifying *H. pylori* ‐negative GC patients, with an AUC of 0.994 (Kim et al. 2024). While integrating different 16S rRNA datasets or additional clinical information can enhance the diagnostic performance of biomarkers, it also increases the complexity of data processing and brings challenges for further validation and practical implementation in clinical settings.

In contrast to individual studies, meta‐analyses have proven to be powerful tools for evaluating combinations of microbial biomarkers to enhance diagnostic accuracy for GC. One meta‐analysis of six independent studies identified a panel of eight biomarkers that distinguished GC patients from non‐GC individuals with an AUC of 0.85 (Liu, Ng, et al. 2022). In 2023, we conducted a meta‐analysis of microbial sequencing data from multiple Chinese cohorts, grouping studies into matched, unmatched, and other categories. This analysis identified broadly applicable microbial biomarkers with high diagnostic accuracy for the Chinese population (Chen et al. 2023). Additionally, another meta‐analysis identified 17 microbial biomarkers with an AUC of 0.908 for differentiating GC from SG and 22 microbial biomarkers with an AUC of 0.964 for distinguishing GC from precancerous lesions (Liu, Zhang, et al. 2022). More recently, Wang, Wang, et al.'s (2024) meta‐analysis showed that a combined model of *Lactobacillus* and *Streptococcus* achieved an AUC of 0.7712 for early GC screening, with further validation yielding AUCs of 0.79 and 0.81. Summarising the results of these four meta‐analyses, three studies consistently identified microbial biomarkers such as *Helicobacter*, *Streptococcus*, *Pseudomonas*, *Selenomonas*, and *Peptostreptococcus*, underscoring their potential relevance in GC diagnosis. These shared biomarkers not only deepen our understanding of the microbial contributions to GC but also lay a solid foundation for the future development of clinically applicable microbial diagnostic tools for GC.

### 3.2 16S rRNA‐Based Microbial Biomarkers for GC Prognosis

The microbiota not only showcases remarkable diagnostic prowess for GC but also harbours substantial potential in prognostic prediction. Presently, a total of one study relying on GM samples and two studies utilising stool samples have explored the GC prognosis via microbial biomarkers. A study evaluating the prognostic value of *Helicobacter*, *Halomonas*, and *Shewanella* in GC reported individual AUC values of 0.684, 0.718, and 0.691, respectively, with a combined AUC of 0.749 (Yang et al. 2023). Likewise, gut microbiota biomarkers such as *Gemmiger*, *Streptococcus*, and *Bacteroides* were identified as predictors for the risk of liver metastasis in GC patients, attaining a maximum AUC of 0.751 (Yu et al. 2021). Additionally, there is evidence indicating that *Roseburia* may potentially predict the risk of peritoneal metastasis in GC patients, with an AUC of 0.698 (Yu et al. 2024). These findings suggest that microbiota‐based biomarkers can be integrated into prognostic models, allowing clinicians to more accurately assess the metastatic potential of GC.

## 4 Potential Pathogenic Mechanisms of Microbial Biomarkers in GC

The above analysis reveals that several microorganisms, namely *Streptococcus*, *Lactobacillus*, *Veillonella*, *Helicobacter*, and *Prevotella*, have been frequently reported in multiple studies and meta‐analyses, demonstrating their diagnostic value for GC. These microorganisms may contribute to the development and progression of GC by influencing DNA damage, oxidative stress, inflammatory responses, immune regulation, and metabolic alterations.

### 4.1 Streptococcus

The recent study has shown that *S. anginosus* can act on the Annexin A2 receptor of gastric epithelial cells through its surface protein *Treponema pallidum* membrane protein C and activate the mitogen‐activated protein kinases (MAPK) signalling pathway, thereby inducing the release of inflammatory factors, disrupting the microbial balance, and forming a pro‐inflammatory microenvironment, which in turn promotes cell proliferation and inhibits apoptosis, further stimulating the development of GC (Fu et al. 2024). These findings provide new insights into the pathogenesis of GC, suggesting that *S. anginosus* may be a potential target for the prevention and treatment of GC in the future. Other studies have shown that co‐infection with *S. salivarius* and *H. pylori* significantly upregulates the expression of pro‐inflammatory cytokines (IL‐1β, IL‐17A, IL‐22, and IFN‐γ), which activate T‐helper 1 (Th1) and Th17 immune responses and recruit a large number of immune cells, particularly FoxP3^+^ regulatory T cells, therefore exacerbating chronic inflammation and promoting the development and progression of GC (Shen et al. 2022). These findings indicate that the synergistic effect amongst microorganisms may further accelerate the progression of cancer and provide new research directions and intervention ideas for the prevention and treatment of GC.

### 4.2 Lactobacillus

*Lactobacillus* species exhibit dual roles in GC. On the beneficial side, strains like *L. plantarum* can inhibit *H. pylori* colonisation and promote GC cell apoptosis by modulating key pathways, including upregulating phosphatase and tensin homologue, Bcl‐2‐associated X, Toll‐like receptor 4, and downregulating protein kinase B (Maleki‐Kakelar et al. 2020). Similarly, *L. casei* induces GC cell apoptosis by suppressing NF‐κB and mammalian target of rapamycin signalling, while *L. acidophilus* NCFM and *L. plantarum* Lp‐115 alleviate *H. pylori* ‐induced inflammation and balance immune responses by reducing Th1 and enhancing Th2 activity (Hwang et al. 2013; Shen et al. 2023). Besides, *Lactobacillus* can also contribute to GC development. It increases lactic acid production to provide energy for tumour cells, reduces nitrates to nitrites to form carcinogenic N‐nitroso compounds that induce epithelial mutations, angiogenesis, and oncogene expression (Vinasco et al. 2019; Li, Liu, et al. 2021). Additionally, it generates reactive oxygen species leading to DNA damage (Jones et al. 2012). Furthermore, the increase in *Lactobacillus* is associated with the rising of CD3^+^ T cells, suggesting it may lead to an imbalance or overactivation of the host immune response, potentially exacerbating the adverse effects on the tumour microenvironment (Qi et al. 2019). The dual role of *Lactobacillus* in GC is closely related to not only strain‐specific characteristics and overgrowth extent but also the multiple regulations amongst its metabolites, the host, environmental factors, and other microbiota.

### 4.3 Veillonella

As a key member of the oral microbiota, *Veillonella* can reduce nitrate to nitrite, leading to nitrite accumulation in the stomach and the formation of carcinogenic N‐nitroso compounds, potentially promoting GC (Bryan et al. 2012; Peng et al. 2017; Park et al. 2022). *V. dispar* also participates in nitrogen metabolism, which is crucial for cancer cell proliferation, further supporting its role in GC development (Kurmi and Haigis 2020; Nath and Natarajan 2024). Given its effect on carcinogenic pathways, understanding and modulating *Veillonella*'s activities could be a promising approach in GC prevention and treatment.

### 4.4 Helicobacter

The classic carcinogenesis caused by *H. pylori* infection has been extensively documented in numerous reviews and research articles. *H. pylori* can induce oxidative stress, DNA damage, and genetic instability through chronic inflammation, virulence factors (cytotoxin‐associated gene A and vacuolating cytotoxin A), and metabolic dysregulation. At the same time, it promotes carcinogenesis and tumour microenvironment formation via immune evasion and gastric mucosal destruction, driving GC progression (Suzuki et al. 2009; Toller et al. 2011; White et al. 2015; Kao et al. 2016; Zhu et al. 2017; Kidane 2018; Salvatori et al. 2023). Additionally, *H. pylori* facilitates GC development by altering the structure of the gastric microbiota. The damage to gastric acid‐secreting glands increases the pH value, which creates a favourable environment for bacteria to produce N‐nitroso compounds, thus promoting GC (Yang et al. 2021). In INS‐GAS mouse models of GC, dysbiosis characterised by a decrease in *Clostridium* and *Bacteroides* and an increase in *Lactobacillus* exacerbates *H. pylori* ‐mediated inflammation, further advancing GC progression (Lertpiriyapong et al. 2014). Furthermore, *S. mutans* in the oral cavity enhances *H. pylori* colonisation in oral and gastric tissues, providing new insights into the synergistic relationship between microbiota and GC (Nomura et al. 2020). This highlights the complexity of GC pathogenesis, making it reasonable to infer that a multi‐target approach addressing both *H. pylori* infection and microbiota dysbiosis may be crucial for more effective prevention and treatment strategies.

### 4.5 Prevotella

*Prevotella* produces a highly efficient redox protein, thioredoxin, which increases oxidative stress and inflammation, contributing to GC development (Abate et al. 2022). At the molecular level, the lipopolysaccharide of *P. intermedia* activates the MAPK signalling pathway to differentiate monocytes into macrophages and release tumour necrosis factor‐α, which triggers inflammatory responses (Liang et al. 2024). In addition, its secreted products upregulate perilipin 3 expression in GC cells, promoting lipid droplet formation and fatty acid oxidation, thereby driving tumour growth (Liang et al. 2024). This underscores the importance of microbial‐mediated inflammation and metabolic reprogramming in cancer progression, suggesting that there may be a potential therapeutic strategy to mitigate GC development by targeting *Prevotella*‐related pathways.

## 5 Problems and Challenges

The 16S rRNA data, with high throughput, culture independence, and high sensitivity and specificity, offer significant advantages in identifying microbiota associated with GC. These attributes make it an indispensable tool for advancing research in this field. However, despite considerable progress, the application of microbial biomarkers in GC still faces several limitations.

### 5.1 The Challenge of Standardising Protocols

Initially, significant differences in microbial communities between GC patients and non‐GC have been observed across different studies. These heterogeneities may be attributed to variations in sample collection and processing methods—such as sampling sites, tissue types of GC cases, storage conditions, and handling procedures—which may result in inconsistencies in identified microbial biomarkers. Moreover, gastrointestinal microbiota, especially for faecal samples, are prone to dietary bias and regional differences, thus underscoring the urgent need to identify more region‐specific and population‐specific microbial biomarkers for broader application across diverse regions and ethnic groups. On the other hand, the GM microbiota has limited diagnostic value, largely due to its composition being significantly influenced by the presence or absence of *H. pylori* infection. When *H. pylori* is present at certain concentrations, it interacts with the mucosal microbiota, altering the overall microbial balance. Therefore, when using the mucosal microbiome for GC diagnosis and prognosis, it is essential to consider the infection status of *H. pylori* . Furthermore, the current differences in sequencing and data analysis methods, such as sequence filtering, clustering algorithms, and classification standards, also hinder the integration of findings across studies. All in all, it is crucial to establish standardised protocols for improving the comparability of research outcomes.

### 5.2 The Challenge From Bench to Bedside

While several diagnostic microbial biomarkers for GC have been identified in recent studies, their transition from bench to bedside remains hindered. The primary obstacles include the small sample sizes used in many investigations that limit statistical efficiency and the lack of rigorous large‐scale external validation to confirm these findings in diverse populations. Without such validation, the generalisability and clinical utility of these biomarkers remain questionable. Although the dynamic changes of the gastrointestinal microbiota accompany the entire process of the development and progression of gastric diseases, current research predominantly focuses on identifying diagnostic biomarkers, with limited exploration of biomarkers for predicting GC prognosis and treatment response. These gaps represent untapped opportunities for advancing precision medicine in GC management, which call for focused research to address multiple clinical needs through developing comprehensive biomarker panels. Lastly, given the strong association between *H. pylori* infection and GC, the impact of *H. pylori* on the composition of the gastric microbiota has been well‐documented. However, in the presence or absence of *H. pylori* infection, microbial biomarkers for the diagnosis, treatment efficacy, and prognosis of GC still require further elucidation.

### 5.3 The Limitation of 16S rRNA Sequencing Technology

In addition, the 16S rRNA technology itself has several inherent limitations. Firstly, 16S rRNA sequencing is a time‐consuming process that involves multiple complex steps, along with the need for specialised equipment and computational resources, thereby limiting its scalability for clinical applications. Furthermore, as this method is based on DNA detection, it cannot differentiate between microorganisms that are alive and metabolically active. This limitation poses a significant challenge in identifying microorganisms that are truly involved in carcinogenesis. Recently, Nikitina et al. (2023) compared DNA sequencing with RNA sequencing to elucidate the composition and function of microbial communities. In contrast to DNA detection, RNA sequencing offers a more dynamic perspective on microbial activity, offering more advantageous means for exploring the relationship between microorganisms and GC. Additionally, 16S rRNA sequencing typically targets specific hypervariable regions (e.g., the V4 region), which only provide information at the genus level and may limit taxonomic resolution. This constraint makes it challenging to distinguish closely related species, potentially underestimating microbial diversity and abundance. Moreover, the technique lacks the ability to directly infer functional and metabolic activities, which hinders a comprehensive understanding of microbial roles. The method also exhibits inherent biases during processing, such as primer bias, contamination, and variability in experimental conditions, which can lead to false positives or negatives, particularly affecting the detection of low‐abundance species. Finally, the accuracy of species annotation, to a large extent, depends on 16S rRNA databases, the update speed of which is often slow, making it challenging to identify newly discovered or poorly characterised species and further restricting the precision and applicability of the technique. Therefore, as emphasised in recent literature, microbiota analysis cannot currently be recommended as a reliable tool for clinical use (Porcari et al. 2025).

## 6 Conclusions and Perspective

The intricate relationship between microbial diversity, composition, and GC underscores the pivotal role of the microbiota in the development and progression of gastric diseases. Despite substantial progress in identifying diagnostic biomarkers, heterogeneities across studies have hindered the formulation of universally applicable conclusions. Current research on prognostic and therapeutic microbial biomarkers remains limited, highlighting the critical gaps that necessitate further investigation to advance the field. Bridging these gaps will require future efforts to focus on more rigorous and standardised study designs, larger and more diverse cohorts, and also a deeper and more comprehensive analysis of interactions amongst gastrointestinal microbiota. Additionally, it is necessary to break through bioactivity detection, resolution enhancement, and functional annotation in order to overcome the inherent limitations of 16S rRNA sequencing. Such advancements will provide a robust foundation for elucidating the mechanism of the microbiota in GC and accelerating the translation of these findings into clinical applications.

## Author Contributions

**Yingying Wang:** writing – original draft, visualization. **Xunan Qiu:** visualization, writing – original draft. **Aining Chu:** writing – original draft. **Jijun Chen:** writing – original draft. **Lu Wang:** writing – original draft. **Xiaohu Sun:** writing – original draft. **Bengang Wang:** conceptualization, supervision, writing – review and editing. **Yuan Yuan:** conceptualization, writing – review and editing, supervision. **Yuehua Gong:** writing – review and editing, supervision, conceptualization.

## Conflicts of Interest

The authors declare no conflicts of interest.

## Data Availability

Data sharing not applicable to this article as no datasets were generated or analysed during the current study.
